# Gene expression profiles and protein-protein interaction networks in amyotrophic lateral sclerosis patients with C9orf72 mutation

**DOI:** 10.1186/s13023-016-0531-y

**Published:** 2016-11-05

**Authors:** Meena Kumari Kotni, Mingzhu Zhao, Dong-Qing Wei

**Affiliations:** 1 College of Life Science and Biotechnology, Shanghai Jiao Tong University, 800 Dongchuan Road, Shanghai, 200240 China; 2 Instrumental Analysis Center, Shanghai Jiao Tong University, 800 Dongchuan Road, Shanghai, 200240 China

**Keywords:** Amyotrophic lateral sclerosis, C9orf72 mutation, Protein-protein interaction network, Hub genes

## Abstract

**Background:**

Amyotrophic lateral sclerosis (ALS) is a neurodegenerative disease that involves the death of neurons. ALS is associated with many gene mutations as previously studied. In order to explore the molecular mechanisms underlying ALS with C9orf72 mutation, gene expression profiles of ALS fibroblasts and control fibroblasts were subjected to bioinformatics analysis. Genes with critical functional roles can be detected by a measure of node centrality in biological networks. In gene co-expression networks, highly connected genes called as candidate hubs have been associated with key disease-related pathways. Herein, this method was applied to find the hub genes related to ALS disease.

**Methods:**

Illumina HiSeq microarray gene expression dataset GSE51684 was retrieved from Gene Expression Omnibus (GEO) database which included four Sporadic ALS, twelve Familial ALS and eight control samples. Differentially Expressed Genes (DEGs) were identified using the Student’s *t* test statistical method and gene co-expression networking. Gene ontology (GO) function and KEGG pathway enrichment analysis of DEGs were performed using the DAVID online tool. Protein-protein interaction (PPI) networks were constructed by mapping the DEGs onto protein-protein interaction data from publicly available databases to identify the pathways where DEGs are involved in. PPI interaction network was divided into subnetworks using MCODE algorithm and was analyzed using Cytoscape.

**Results:**

The results revealed that the expression of DEGs was mainly involved in cell adhesion, cell-cell signaling, Extra cellular matrix region GO processes and focal adhesion, neuroactive ligand receptor interaction, Extracellular matrix receptor interaction. Tumor necrosis factor (TNF), Endothelin 1 (EDN1), Angiotensin (AGT) and many cell adhesion molecules (CAM) were detected as hub genes that can be targeted as novel therapeutic targets for ALS disease.

**Conclusion:**

These analyses and findings enhance the understanding of ALS pathogenesis and provide references for ALS therapy.

**Electronic supplementary material:**

The online version of this article (doi:10.1186/s13023-016-0531-y) contains supplementary material, which is available to authorized users.

## Background

Amyotrophic lateral sclerosis (ALS) is a neurodegenerative disease that involves the death of neurons [1, 2]. ALS is often called Lou Gehrig’s disease, after the famous baseball player who was diagnosed with it. The occurrence of ALS is two per 100,000 people, and it is estimated that more than 20,000 Americans may be living with ALS at any given time. It is worldwide with no racial, ethnic or socioeconomic boundaries and can affect anyone. It affects the nerve cells in the brain and the spinal cord and is characterized by stiff muscles, muscle twitching and gradually worsening weakness due to muscles decreasing in size. Despite intensive research, the clinical and pathophysiological mechanisms of ALS still remain unclear. There are two types of ALS, sporadic (SALS) and familial (FALS). 90–95 % of ALS cases are sporadic for which the cause is unknown and 5–10 % are familial, which are inherited [3]. Findings on ALS patients have drawn in numerous genes related to ALS pathogenesis and have identified diverse processes, such as excitotoxicity, oxidative stress, cytoskeletol abnormalities, impaired axonal transport, mitochondrial dysfunction and protein aggregation. Presently, ten genes such as SOD1, ALSIN, SETX, SPG11, FUS, VAPB, TARDBP, OPTN, ATXN2 and C9orf72 were found to affect the pathogenesis of ALS [4]. Though SALS and FALS are clinically and pathophysiologically similar, there is a requirement of finding the genes involved in FALS and the molecular mechanism pathways involved in the mutation of these genes. Till date studies were done to know the molecular mechanisms and underlying pathogenesis of ALS disease related to SALS with Superoxide dismutase 1 (SOD1) mutation [5, 6]. As the study of SOD1 has led to great advances in proper illustration of molecular mechanisms underlying in ALS disease, identifying the mutations in other genes and the pathways involved in these mechanisms is utmost important. Till date there is no cure for ALS, however the drug riluzole – the only prescribed drug approved by Food and Drug Administration (FDA) to treat ALS which prolongs the life by 2–3 months but do not relieve the symptoms [7, 8]. Improving diagnoses and treatment of this disease is utmost essential, as currently there exists no cure for ALS.

C9orf72 mutation is currently the major genetic cause of ALS disease accounting for approximately 34.2 % of the familial ALS cases. C9orf72 mutation is a hexanucleotide repeat expansion of the six letter string of nucleotides GGGGCC. Although nothing about C9orf72 function [9, 10] and the mechanisms by which expanded repeats cause neurodegeneration are known, C9orf72 RNAs levels were found to be reduced as reported in tissue samples from patients with C9orf72 expansions [11–13]. The leading candidate mechanisms by which expanded repeats cause neurodegeneration are RNA-mediated toxicity, loss of C9orf72 gene function, or a combination of the two. In a normal person there would be few repeats of this nucleotide, but in the person with C9orf72 mutation this repeat can occur in the order of hundreds that result in the formation of RNA foci in white blood cells, fibroblasts, glia and multiple neuronal cell types. These RNA foci are not present in the sporadic ALS and familial ALS caused by other mutations. This RNA-mediated toxicity is found to play a crucial role in a variety of repeat expansion disorders by accumulation of expanded transcripts into nuclear RNA foci [14]. These RNA repeats fold into stable structures and sequester RNA binding proteins to set off a molecular cascade leading to neurodegeneration [14]. A loss of C9orf72 gene function is observed in patients with expansion by reported reductions of C9orf72 transcript levels [11–13] Although this reduction to neuronal death is not established, loss of C9orf72 gene function during embryonic development is associated with motor deficits in zebrafish [15].

Lagier-Tourenne et al. have demonstrated the ASO mediated reduction of C9orf72 RNAs by aggravating the loss of C9orf72 function [16]. In this view of finding different molecular mechanisms and pathways related to C9orf72 mutation in ALS disease, we have used systems biology approach for the gene expression data to identify Gene Ontologies (GO) and pathways related to familial ALS. Genome-wide RNA profiling in fibroblasts from patients with C9orf72 expansion was studied in the RNA microarray data [9] used for the present study. This microarray data retrieved from Gene expression omnibus (GEO) [17] was screened to identify differentially expressed genes (DEGs) between ALS and control fibroblasts before and after ASO mediated disease therapy in order to find potential genes for the pathogenesis of ALS. Gene ontology(GO) enrichment analysis and Kyoto encyclopedia of Genes and Genomes(KEGG) pathway analysis was performed, and a protein-protein interaction (PPI) network was constructed by mapping the DEGs to the human PPI data. Our research laid a bridge between the DEGs, the KEGG pathways and the protein-protein interaction networks which will help for the development of novel targets for ALS therapeutic intervention.

## Methods

### DEGs analysis and Co-expression network

Illumina HiSeq microarray dataset (GSE51684) [16] with their RPKM (Reads per kilo base per million mapped reads) values was retrieved from GEO (http://www.ncbi.nlm.nih.gov/geo/) [17] which was based on the GPL 11154 Illumina HiSeq 2000 platform, having four sporadic ALS, twelve familial ALS and eight control samples. Familial ALS samples and the control samples were used for the present study to identify the hub genes that can act as probable familial ALS targets. Samples were divided into six groups (Table 1) based on a) presence/absence of C9orf72 mutation and b) treatment of the disease with the control/C9orf72 Antisense oligonucleotide (ASO). The four Familial ALS samples having C9orf72 mutation (Group III) and four control samples (Group VI) without ASO treatment were put into network 1 and four familial ALS samples (Group II) and four control samples (Group IV) treated with the C9orf72 ASOs were put into network 2. All the genes with RPKM > 5 in atleast one sample were taken for finding DEGs. The software R v 3.2.2 [18] was used to perform all the statistical analyses. The Student’s *t* test statistical *P*-values and fold changes were calculated. Further, each *P*-value is adjusted with a Benjamini-Hochberg method to account for multiple testing. The Benjamini-Hochberg method provides sufficiently conservative estimates of “significance” among the many statistically detectable scores. Genes with fold change > 2.0 and < 0.5 and the adjusted *P*-value < 0.05 were identified in both the networks (Additional file 1: Table S1). Gene co-expression network analysis was performed by constructing a matrix of pairwise Pearson correlations between all genes identified by statistical methods across all selected samples. Finally co-expression threshold of > 0.9 was set to find the DEGs in both the networks. This study aimed at obtaining the DEGs for C9orf72 ASO treated samples over ASO untreated samples.

**Table 1:** Classification of samples into groups based on genotype and ASO treatment

S.No	Group No.	No. of samples	Genotype	Treatment
1.	Group I	4	C9orf72expansion	CTRL ASO
2.	Group II	4	C9orf72expansion	C9orf72 ASO
3.	Group III	4	C9orf72expansion	No treatment
4.	Group IV	4	Non-neurologic control	C9orf72 ASO
5.	Group V	4	Sporadic ALS	No treatment
6.	Group VI	4	Non-neurologic control	No treatment

### Enrichment analysis of GO function and KEGG pathway

The information on the networked molecules and genes is contained in the KEGG. The database for annotation, visualization and integrated discovery (DAVID) was used to analyze list of genes derived from high-throughput genomic experiments. DAVID online tool [19] for Gene ontology (GO) annotations and KEGG pathway analysis were used to perform the enrichment analysis of the biological processes of DEGs in order to identify the enriched genes at the cellular level. The cut-off criteria of more than two genes, FDR and *P*-values less than 0.05 were chosen.

### Construction of gene/protein interaction network and analysis

Human protein – protein interaction network (PPI) data were obtained from public databases MINT [20], BioGrid [21] and HPRD [22]. Potential PPI correlations were demonstrated by mapping all the DEGs on the compiled data set of human interactome for the PPI network construction and microarray data enrichment analysis. The DEGs showed to have 1885 interactions reported in the databases and visualized in CytoHubba [23]. Scale-free property of the protein interaction network was used to find the key hub proteins. PPI network was constructed based on the PPI correlations by the Cytoscape v3.2.0 software platform.

### Molecular complex detection analysis

The molecular complex detection (MCODE) algorithm [24] is a well known automated method using the Cytoscape MCODE plug-in to find highly interconnected subgraphs or modules that detects densely connected regions in large PPI networks that may represent molecular complexes. In the present study, Cytoscape MCODE plug-in was used to search clustered subnetworks of highly intraconnected nodes (n > 15). Then the identified modules were used for functional enrichment analysis using the BinGO [25] plug-in of Cytoscape. Validation of molecular mechanism of ALS and finding potentially essential genes can be performed through these analytical results.

## Results

### DEGs analysis and Co-expression network

The two networks constructed were used to find the DEGs that could be probable targets for familial ALS disease. In the present study network 1 and network 2 were compared to find highly expressed genes before and after ASO treatment of C9orf72 fibroblasts and control fibroblasts. Statistical analysis has yielded 1055 DEGs. Of these, 734 genes were upregulated and 321 genes were downregulated (Additional file 1: Table S1). Statistical methods used in the present study could identify more number of DEGs when compared to the DEGs reported by Lagier-Tourenne et al. [16]. Several important DEGs were identified, including Tumor necrosis factor (TNF), Endothelin 1 (EDN1), Angiotensin (AGT), Apolipoprotein E (APOE), Vitronectin (VTN), Von Willebrand factor (VWF), Thrombospondin receptor (CD36), TNF Receptor Superfamily Member 5 (CD40), Integrin, Alpha 3 (ITGA3), Integrin, Alpha 7 (ITGA7), Transforming growth factor beta 2 (TGFB2), Aggrecan (ACAN),Wingless-Type MMTV Integration site family, member 5A (WNT5A), Neurotrophic tyrosine kinase, receptor, type 2 (NTRK2) and Zinc finger protein GLI1 (GLI1) which were involved in the biological processes related to ALS.

### GO function and KEGG pathway enrichment analysis

Functional annotation of large-scale genomic data can be obtained by GO analysis approach. The DEGs were mapped to the DAVID database to investigate the functional changes in the ALS diseased patients. DAVID is the most popular tool in the field of high-throughput functional annotation that is cited in more than 2000 publications. The top GO enrichment analysis results of DEGs based on biological process, cellular components and molecular function are shown in Table 2. According to the biological processes analysis, upregulated DEGs were mainly involved in cell adhesion, biological adhesion, cell-cell adhesion and cell-cell signaling. Based on the molecular function analysis, DEGs were involved in calcium ion binding activity. In addition, upregulated DEGs were located in the extracellular matrix (ECM), extracellular space, extracellular region part. The downregulated DEGs were enriched only in the extracellular region part with FDR 3.3E-08.

**Table 2:** Top GO terms significantly enriched with high counts of DEGs in samples from ALS fibroblasts and control fibroblasts

Term	Category	Count	*P*-value	FDR value	Description
GO:0007155	BP	87	2.4E-16	2.2E-09	Cell adhesion
GO:0022610	BP	87	2.6E-16	2.3E-09	Biological adhesion
GO:0007267	BP	65	8.4E-10	3.4E-04	Cell-cell signaling
GO:0007267	BP	39	3.2E-09	2.3E-04	Cell-cell adhesion
GO: 0005576	CC	200	2.1E-23	2.9E-10	Extracellular region
GO:0044421	CC	123	1.4E-22	1.3E-10	Extracellular region part
GO: 0031012	CC	57	4.6E-15	2.9E-06	Extracellular matrix
GO: 0005615	CC	81	9.6E-13	1.9E-06	Extracellular space
GO:0005509	MF	100	4.2E-15	2.3E-08	Calcium ion binding

Different pathways were identified in the KEGG pathway enrichment analysis as shown in Table 3. Upregulated DEGs were mainly involved in ECM-receptor interaction, focal adhesion, Cell adhesion molecules (CAMs) whereas the downregulated genes were not enriched in any of the KEGG pathways.

**Table 3:** Top four enriched KEGG pathways of DEGs with low *P*-values in samples from ALS fibroblasts and control fibroblasts

KEGG	Count	*P*-value	FDR value	Description
KEGG_PATHWAY	17	2.0E-06	3.8E-03	Extracellular matrix(ECM)-receptor interaction
KEGG_PATHWAY	24	9.8E-05	3.7E-02	Focal adhesion
KEGG_PATHWAY	17	6.1E-04	2.7E-02	Cell adhesion molecules (CAMs)

### PPI network construction

**Fig. 1:**
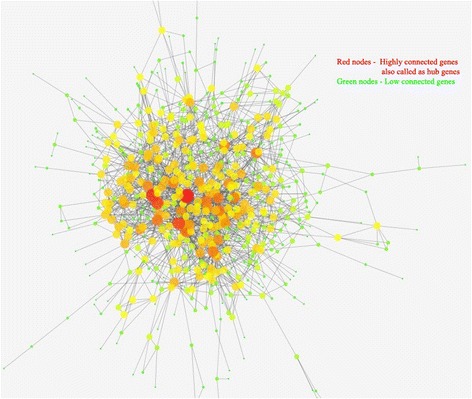
PPI network constructed from DEGs on the basis of human interactome (Nodes with higher degree values (hubs) are depicted with large shape and red colour with continuous gradient mapping to green colour with lower degree value)

**Fig. 2:**
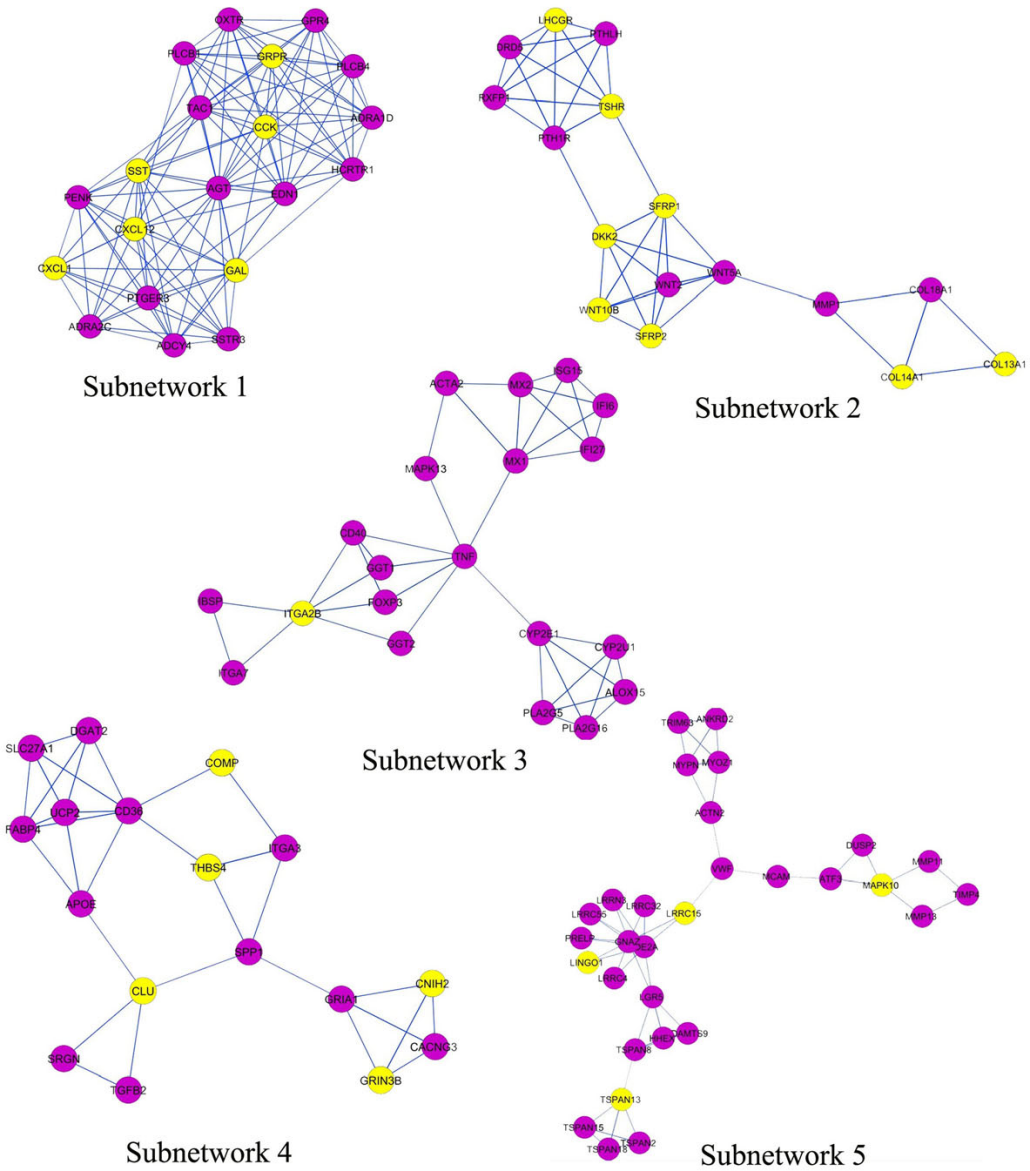
Subnetworks identified from PPI of DEGs. Nodes in violet color are upregulated genes, yellow nodes are downregulated genes and blue lines are interactions between nodes

PPI network analysis has been a powerful tool for understanding the biological responses in health and disease. In the PPI network, the protein is defined as the node and the interaction between two nodes is defined as the edge. The DEGs related to ALS were mapped onto the reference network obtained from Mint, HPRD and BioGrid databases (Fig. 1) where the red colour nodes indicate highly connected ‘hub’ nodes in PPI network. The resulting network was divided into subnetworks each of which represented protein subcomplexes or functional modules. This subnetworking would provide more specific and detailed information about the PPI network. The subnetworks were obtained using the MCODE plug-in implementing the MCODE algorithm with the k-score value of 2.0, node score cutoff of 0.2, maximum depth from the seed node of 100 and graphics-processing-unit-based parallelization to find modules efficiently. A total of 19 modules were found, among which five modules were detected with the intra-connection nodes >15 and node score >2.0 (Table 4, Fig. 2). All clustered modules varied in size comprising a total of 103 proteins and 263 PPIs.

**Table 4:** Statistics for top five subnetworks identified by MCODE method in PPI network

Subnetwork	Score	Proteins	Interactions
1.	12.105	20	115
2.	5.067	16	38
3.	4.105	20	39
4.	3.875	17	31
5.	3.379	30	40

Next, the complete PPI network was visualized in Cytohubba to find the hub genes with high degree of connectivity between the nodes. The higher value for the degree indicate highly connected network and likely to be more robust. The degree of each node in the network was calculated by Cytohubba, which identified hubs as nodes with the degree value >25 (Table 5).

**Table 5:** Top 15 hub nodes identified in PPI network for DEGs from ALS fibroblast samples and control fibroblast samples

Gene	Gene Name	Degree	GO Term
TNF	Tumor necrosis factor	47	Celladhesion/Cell-cell adhesion/ECM
EDN1	Endothelin 1	42	Cell-cell signaling/ECM
AGT	Angiotensin	37	Celladhesion/Cell-cell signaling/ECM
WNT5A	Wingless-Type MMTV Integration site family, member 5A	31	Cell-cell signaling/ECM
APOE	Apolipoprotein E	29	Cell-cell signaling
VWF	Von Willebrand factor	28	Cell adhesion/ECM
ACAN	Aggrecan	24	Celladhesion/Cell-cell adhesion/ECM
CD36	Thrombospondin receptor	24	Cell adhesion
VTN	Vitronectin	23	Cell adhesion/ECM
CD40	TNF receptor super family member 5	25	Cell adhesion molecules/Extracellular region
NTRK2	Neurotrophic tyrosine kinase, receptor, type 2	23	Cell-cell signaling
ITGA3	Integrin, alpha 3	21	Cell adhesion/focal adhesion
ITGA7	Integrin, alpha 7	21	Cell adhesion/focal adhesion
GLI1	Zinc finger protein GLI1	19	Cell-cell signaling
TGFB2	Transforming growth factor beta 2	19	Cell-cell signaling

Finally, functional annotation of submodules was predicted. Most of the hub genes as mentioned in Table 5 were involved in the subnetworks indicating the reliability of the subnetwork model generated and these hub genes were primarily corresponding to adhesion, signaling and Extra cellular matrix as according to the results obtained from GO enrichment analysis and KEGG pathways with *P*-values and FDR in the acceptable range.

Subnetworks 1, 2, 4 and 5 were enriched in the GO terms related to the chemical component extracellular region part, extracellular matrix. Subnetworks 1 and 2 were enriched in biological process GO term related to cell-cell signaling. Subnetwork 4 was enriched in the biological process related to cell adhesion and biological adhesion. Subnetwork 3 was not enriched in any of the GO term. The *P*-values of all the enriched GO terms were in the range of 5.4E-17 to 8.9E-03.

KEGG pathway neuroactive ligand receptor interaction was enriched in Subnetworks 1 and 2. ECM receptor interaction pathway was enriched in subnetworks 3 and 4. Subnetwork 5 was enriched in focal adhesion pathway. The *P*-values of all the enriched KEGG pathways were in the range of 3.1E-05 to 2.1E-03.

## Discussion

In this study, we used the Human Illumina microarray data to find hub genes associated with C9orf72 mutation in FALS disease. C9orf72 expansion was found to be the most common genetic abnormality in FALS [26]. The discovery of the expansion repeat in C9orf72 is too recent [27] to know its complete relationship with the inflammatory system. This repeat expansion has opened new avenues of research in ALS. A total of 1055 DEGs in the C9orf72 patient fibroblasts were identified compared to the control fibroblasts. We could find more number of DEGs compared to the DEGs reported by Lagier-Tourenne et al.[16]. The DEGs were mainly enriched in the GO terms related to cell adhesion, biological adhesion and cell-cell signaling biological processes, extracellular matrix and extracellular space cellular components and the calcium ion signaling molecular function. The pathways enriched by the DEGs are ECM receptor interaction pathway, focal adhesion pathway, cell adhesion molecules. 15 hub genes were identified in the PPI network that was associated with the GO terms and pathways enriched by the DEGs (Table 5).

Tumor necrosis factor (TNF), Endothelin 1 (EDN1) were detected as the main hubs with a degree value of >35. TNF is a pro-inflammatory cytokine produced by monocytes and activated by mast cells, fibroblasts and neurons during acute inflammation. TNF is known to be secreted by the brain resident macrophage i.e. the microglial cell in response to various stimuli and its over production is related to neuronal cell death [28]. Elevated production of TNF is a common feature of several inflammatory diseases including ALS. It is known to play a major role in the central nervous system (CNS) neuroinflammation-mediated cell death in ALS as well as several other CNS complications. As a consequence of innate immune activation, an increased level of TNF is observed in many neurodegenerative diseases as reported earlier [29–32]. Previously, FALS and SALS affected spinal cord of mice was investigated and found increased immunoreactivity for TNF. Inhibiting the expression of TNF was observed in the ALS affected mice treated with thalidomide and lenalidomide drugs. This result gave the confirmation of the hypothesis that TNF plays an important role in the pathogenesis of ALS disease [33, 34]. The overexpression of TNF is observed in the ALS disease fibroblast samples taken for the present study with the fold change of 4.5 compared to the control fibroblast samples. TNF is involved both in immunological pathways and in oxidative stress known in ALS disease [35–37]. Hence modulating the levels of TNF would be worthwhile therapy for various CNS related diseases making TNF as the therapeutic target for neurodegenerative diseases including ALS disease.

EDN1 and its receptor B have been previously reported to be associated with ALS pathology [38]. It is a pro-inflammatory vasoconstrictor peptide encoding gene known to exert a variety of ALS-aggravating effects that includes axonal degeneration [39], heightened sensitivity to hypoxic stress [40] and increased excitotoxicity [41]. The expression of EDN1 in astrocytes and microglia is required for their survival under oxidative stress [42] which is a central mechanism by which motor neuron death occurs. EDN1 is found to be abundantly expressed by reactive astrocytes in the spinal cord of mouse model and SALS patients [43]. Several experiments have been performed to understand the toxic effects and lower the levels of EDN1 that would affect the motor neuron cell death. These experiements would suggest EDN1 as the potential target for therapeutic invention in ALS [43].

Furthermore, GO functional analysis also demonstrated that the DEGs were mainly involved in cell adhesion, biological adhesion, cell-cell signaling based on the analysis of biological processes. Adhesion plays an important role in cell signaling, regulation and fundamental in the development and maintenance of tissues [44].

Progression of ALS disease was known to involve cell-to-cell transmission involving motor neurons, microglia and astrocytes [45, 46].

Substantial clinical and pathological characteristics overlap among the common neurodegenerative diseases, ALS, Frontotemporal dementia (FTD) and Alzheimer’s disease (AD). C9orf72 repeat expansions were found to be risk factors for ALS, FTD and AD [47, 48]. Kohli et al. and Beck et al. demonstrated the risk factors of AD to be C9orf72 repeat expansions [49, 50].

Cell adhesion is involved in many aspects of neuronal development, including axon-bundle formation, synapse formation and formation of glial networks that surrounds axons and synapses [51–53]. The mechanical and chemical linkages between the intracellular and extracellular space are formed by cellular adhesions [54]. These adhesion systems forms the major for brain morphology and highly coordinated brain functions, such as memory and learning [51–53]. Various cell adhesion molecules (CAMs) are involved in the synapse formation and neuron-glia interactions [54]. These adhesions makes the interactions between the cells and the ECM through the cell adhesion molecules which also involved in the cell migration and signaling of many biological processes. This functionality of adhesion and adhesion molecules has made this a topic of study in the present scientific community. Previously reports stated cell adhesion molecules (CAMs) as disease biomarkers or pharmaceutical targets of neurological diseases [55].

The upregulated genes with highest hub degree mainly TNF, AGT, VWF, ACAN, VTN, ITGA3, ITGA7 are enriched in the cell adhesion biological process which forms the basis for neuronal damage or repair. The cell adhesion molecules (CAMs) ITGA3, ITGA7, CD36 and CD40 are differentially expressed which makes the links between the cells and ECM for cell migration and signaling cascades of many biological processes. Cell adhesion had previously been shown to be related to neurodegenerative diseases [56] and the CAMs were involved in the pathogenesis of many neurodegenerative diseases as previously reported [57, 58]. These CAMs play an important role in the inflammatory mechanisms associated with neurodegeneration [59]. Specific ECM molecules bind to integrin cell surface receptors and lead to downstream focal adhesion activation involved in the regulation of cell survival signals. Integrins and focal adhesion CAMs’ studies indicate a role for signaling in neurite outgrowth differentiation and in response to the toxic effects associated with neurodegeneration [60, 61].

Neuro active ligand receptor interaction KEGG pathway is highly enriched in the subnetworks. Many GPCRs are involved in this pathway. Signal transduction pathways activated by GPCRs are known to regulate variety of neuronal functions, plasticity and synaptic transmission, and control of various behaviours including memory, learning, emotions and motor functions. Most of the neurological disorders involve degeneration and death of neurons in brain and spinal cord. For example, ALS is characterized by selective degeneration of motor neurons in the spinal cord. The causes of neurodegenerative conditions are different in different diseases, but they do share mechanisms that include metabolic compromise, oxidative stress and disruption of cellular calcium homeostasis [62–64]. GPCRs are expressed in neurons and/or glial cells in central nervous system affected with neurodegenerative diseases. Increasing evidences suggest that signaling pathways play an important role in modifying the neurodegenerative processes.

Previous studies have reported TNF as the therapeutic target for neurodegenerative [65] and also CAMs have been reported as targets for many neurodegenerative diseases [59]. All these forms the important aspects of the central nervous system hence could be important therapeutic targets for neurodegenerative diseases including ALS.

## Conclusion

In the present study, systems biology approach was used to examine the relationships between the importance of genes and several topological characteristics in the human PPI network. There are many studies previously reported with the mutations and pathways associated with SOD1 gene mutation and the pathways involved in its pathogenesis. We have uncovered gene-expression changes that occurred in familial ALS with C9orf72 mutation with ASO mediated reduction of C9orf72 RNAs. In view of this, gene expression data of C9orf72 fibroblasts and control fibroblasts were used to identify the DEGs related to ALS. Various bioinformatic tools were used to find the hub genes, enriched GO terms and KEGG pathways. GO terms related to adhesion, signaling were the main terms enriched by the DEGs. TNF, EDN1, AGT along with many other CAMs were identified to be the possible potential genes as targets for ALS disease. However, further studies are required to determine the clinical utility of these observations in the therapeutic management of ALS related neurological disease.

## Additional file

Additional file 1: Differentially Expressed Genes in C9orf72 patients compared to control individuals fibroblasts. (XLSX 74 kb)

## Abbreviations

ACAN: Aggrecan
AD: Alzheimer’s disease
AGT: Angiotensin
ALS: Amyotrophic lateral sclerosis
APOE: Apolipoprotein E
ASO: Antisense oligonucleotide
CAM: Cell adhesion molecule
CD36: Thrombospondin receptor
CD40: TNF receptor superfamily member 5
CNS: Central nervous system
DAVID: Database for annotation, visualization and integrated discovery
DEG: Differentially expressed genes
ECM: Extracellular matrix
EDN1: Endothelin 1
FALS: Familial amyotrophic lateral sclerosis
FDA: Food and drug administration
FTD: Frontotemporal dementia
GEO: Gene expression omnibus
GLI1: Zinc finger protein GLI1
GO: Gene ontology
ITGA3: Integrin, alpha 3
KEGG: Kyoto encyclopedia of genes and genomes
MCODE: Molecular complex detection
NTRK2: Neurotrophic tyrosine kinase, receptor, type 2
PPI: Protein-protein interaction
SALS: Sporadic amyotrophic lateral sclerosis
SOD1: Superoxide dismutase 1
TGFB2: Transforming growth factor beta 2
TIGA7: Integrin, alpha 7
TNF: Tumor necrosis factor
VTN: Vitronectin
VWF: Von willebrand factor
WNT5A: Wingless-type MMTV integration site family, member 5A.
